# Roots of angiosperm formins: The evolutionary history of plant FH2 domain-containing proteins

**DOI:** 10.1186/1471-2148-8-115

**Published:** 2008-04-22

**Authors:** Michal Grunt, Viktor Žárský, Fatima Cvrčková

**Affiliations:** 1 Department of Plant Physiology, Faculty of Sciences, Charles University, Vinièná 5, CZ 128 43 Praha 2, Czech Republic; 2 Institute of Experimental Botany, Academy of Sciences of the Czech Republic, Rozvojová 135, CZ 165 02 Praha 6, Czech Republic

## Abstract

**Background:**

Shuffling of modular protein domains is an important source of evolutionary innovation. Formins are a family of actin-organizing proteins that share a conserved FH2 domain but their overall domain architecture differs dramatically between opisthokonts (metazoans and fungi) and plants. We performed a phylogenomic analysis of formins in most eukaryotic kingdoms, aiming to reconstruct an evolutionary scenario that may have produced the current diversity of domain combinations with focus on the origin of the angiosperm formin architectures.

**Results:**

The Rho GTPase-binding domain (GBD/FH3) reported from opisthokont and *Dictyostelium *formins was found in all lineages except plants, suggesting its ancestral character. Instead, mosses and vascular plants possess the two formin classes known from angiosperms: membrane-anchored Class I formins and Class II formins carrying a PTEN-like domain. PTEN-related domains were found also in stramenopile formins, where they have been probably acquired independently rather than by horizontal transfer, following a burst of domain rearrangements in the chromalveolate lineage. A novel RhoGAP-related domain was identified in some algal, moss and lycophyte (but not angiosperm) formins that define a specific branch (Class III) of the formin family.

**Conclusion:**

We propose a scenario where formins underwent multiple domain rearrangements in several eukaryotic lineages, especially plants and chromalveolates. In plants this replaced GBD/FH3 by a probably inactive RhoGAP-like domain, preserving a formin-mediated association between (membrane-anchored) Rho GTPases and the actin cytoskeleton. Subsequent amplification of formin genes, possibly coincident with the expansion of plants to dry land, was followed by acquisition of alternative membrane attachment mechanisms present in extant Class I and Class II formins, allowing later loss of the RhoGAP-like domain-containing formins in angiosperms.

## Background

Domain shuffling in modular proteins is considered one of the major sources of evolutionary innovation [1]. Phylogenetic studies of signaling and regulatory proteins exhibiting variable domain composition can provide important contributions towards understanding the basis of the current diversity of life.

Formins (or FH2 domain-containing proteins) may serve as a good example of an ancient protein family with a likely role in morphogenesis (at least on the cellular level) whose evolution included extensive domain rearrangements. The well-conserved FH2 (formin homology 2) domain [Smart: SM00498, Interpro: IPR015425, Pfam: PF02181] can nucleate new actin filaments by a unique „leaky barbed-end cap“ mechanism, and in some situations acts as a capping protein (for a review see [2,3]). Besides FH2, most formins possess a N-terminally located proline-rich region (termed the FH1 domain), implicated in interactions with the actin monomer-binding protein profilin. Some formins also share additional („optional“) domains that vary substantially between diverse eukaryotic lineages.

Metazoan formins often contain a N-terminal GTPase binding domain (GBD, [Pfam: Drf_GBD]) interacting with Rho class GTPases. GBD usually overlaps with another conserved domain, FH3 [Pfam: Drf_FH3], and the GBD/FH3 motif is recognized by a C-terminally located autoinhibitory domain (DAD – Diaphanous autoregulatory domain, [Pfam: Drf_DAD]). This domain architecture, which is believed to mediate control of the formin's actin-nucleating activity by Rho-related GTPases [4], was originally considered characteristic for metazoan diaphanous-related formins but later found to be widespread, though not ubiquitous, not only in metazoans, but also in fungi and *Dictyostelium *[5].

FH2-containing proteins tend to form extensive families of paralogs (mouse, e.g., has at least 15 formin-encoding genes that can be assigned to 7 conserved classes [6]), and their overall domain organization is often variable within the repertoire encoded by any given genome. Nevertheless, certain domain arrangements are more frequent, present in multiple species of a given taxon, and therefore probably ancestral. This is apparently also the case of the GBD/FH3-FH1-FH2-DAD architecture.

However, no GBD/FH3-containing formins have been identified in plants so far. Formins from angiosperms can be unequivocally assigned to one out of two classes, based on primary structure of their FH2 domains [7,8]; none of them is orthologous to any of the proposed seven metazoan formin classes [6], or to fungal formins.

Each angiosperm formin class exhibits also a characteristic domain composition. The „prototype“ structure for plant Class I formins includes a N-terminal membrane insertion signal, followed by a supposedly extracytoplasmic Pro-rich stretch, a transmembrane region, and C-terminal FH1 and FH2 domains [9]. Experimental studies confirmed membrane association for several Class I formins in *Arabidopsis *and suggested within-class functional differences reflected e.g. in varying tissue- or organ-specific expression patterns or in protein localization to specific subcellular destinations [10-15]. A novel mode of interaction with actin has been documented for a Class I member, AtFH1 from *Arabidopsis*, which can induce formation of filament bundles in addition to formin nucleation [16].

Angiosperm Class II formins usually (though not always) carry a N-terminal domain related to members of another conserved protein family whose founding member is the human antioncogene PTEN, recently implied also in the pathogenesis of the Parkinson disease (reviewed in [17]). The conventional PTEN domain exhibits a phosphatase activity towards both lipids and proteins and is believed to mediate lipid-based signaling affecting e.g. actin organization, cytokinesis and development of cell surface structures in organisms as diverse as the human, *Drosophila *and *Dictyostelium *[18-21]. Surprisingly, the phosphatase catalytic site is eroded by mutations in the PTEN-like domains of plant formins, suggesting that this domain may perhaps participate in localization of the FH2 domain rather than exhibiting its own catalytic activity [8].

PTEN-related domains are found also in metazoan tensin (a multifunctional protein involved in integrin-mediated focal adhesions and in cell motility, which can also cross-link actin filaments and cap their barbed ends [22-24]), auxilins (proteins participating in uncoating of clathrin-coated vesicles) and in the auxilin-like domain of the cyclin G-associated protein kinase [22,25], indicating that the PTEN domain is, like FH2, a versatile building block capable of entering into multiple contexts.

It is therefore not surprising that PTEN family proteins without any obvious relationship to the formins have been found also in plants. *Arabidopsis *has three PTEN homologues, one of them (AtPTEN1) essential for the male gametophyte development [26]; the phenotype of the non-viable mutant pollen suggests an involvement in cell surface organization.

Systematic phylogenetic studies of the formin repertoire encoded by complete genomes have been so far restricted on one hand to the opisthokont lineage (Metazoa and Fungi) and the social amoeba *Dictyostelium discoideum *[5,6] which may be relatively close to opisthokonts [27], or, on the other hand, to vascular plants, predominantly angiosperms [7,8]. Thus, until now, we could not decide whether any of the opisthokont (or plant) formin architectures represents either a conserved ancestral state or a late invention. Using the growing thesaurus of publicly available sequence data, we have attempted to map the formin diversity across five out of the six major eukaryotic kingdoms (*sensu *[27]), with particular attention to plants. The results yielded a possible evolutionary scenario that may have produced the extant domain architecture of plant formins, and provided interesting insights into the evolutionary dynamics of modular regulatory proteins in general.

## Results

### A collection of eukaryotic formin sequences

To obtain a broader view of the formin diversity, we scanned 36 available complete or nearly complete eukaryotic genome sequences, as well as EST and cDNA databases of additional eleven species, using known representatives of plant Class I and Class II, metazoan and yeast formins as queries (see Materials and Methods). As a result, we have assembled a collection of 122 plant formin sequences from 16 species (including selected non-seed plants), as well as 173 formins from 31 species of the metazoans, fungi, amoebae, chromalveolates and excavates (see Additional file 1 for a full list of species and genes and Additional file 2 for newly predicted or revised protein sequences). As expected, all complete genomes studied encoded at least one FH2-containing protein, supporting the notion that the FH2 domain belongs to a set of proteins present already in the last common ancestor of eukaryotes. However, we were unable to find any possible prokaryotic relatives, i.e. bacterial or archeal proteins exhibiting significant similarity to the FH2 domain.

Somewhat surprisingly, we have noticed that considerable expansion of the FH2 protein family is not restricted to multicellular organisms. In contrary, nearly all complete genomes analyzed (except three ascomycete fungi whose genomes are still at a draft stage) encoded at least two FH2-containing proteins, and even unicellular organisms such as the excavate *Naegleria gruberi *(a flagellate amoeba) or the ciliate *Paramecium tetraurelia *possess 15 or 14 formins, respectively – i.e. a number comparable to that reported previously for mammals [6] or flowering plants [8].

### Phylogeny of the FH2 domain: evidence for multiple independent gene duplications

**Figure 1:**
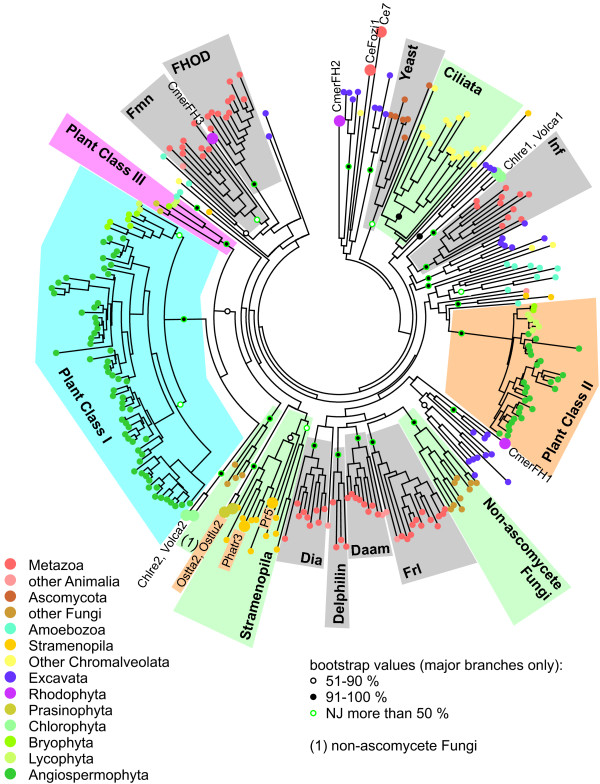
**Overview of FH2 domain phylogeny**. A schematic summary of an unrooted maximum likelihood tree based on 287 FH2 domain sequences (for a full tree see Additional file 3, for a full list of genes and species, see Additional file 1). The remaining 8 FH2 sequences from our collection were either incomplete or found only during the final database checks; for these cases, closest relatives are shown in Additional file 1. Symbols at branches denote percentual bootstrap values (out of 500 replicates; shown for major branches only). Branches supported also by at least 51% of bootstrap replicated in a tree constructed using the NJ method are denoted by green symbols. Clades on a gray background correspond to previously known metazoan or fungal formin classes, clades on a green background to novel multi-species formin clades outside plants (a non-ascomycete fungi group encompassing Phybl1-3, Phybl5-7, Pospl2 and Um2; a stramenopile group including Tp1-6, Phatr1-4 and Pr5, 6; a ciliate clade consisting of Pt1-10 and Tt1-3). Gene name abbreviations are shown for PTEN-containing formins outside plant Class II (sand-colored background) and for yeast and metazoan outliers.

The apparent widespread multiplication of formin-encoding genes in diverse lineages raises the question whether any of the extant formins can be assigned to well-defined groups of orthologues beyond those identified in previous phylogenetic studies [5,6,8]. We have thus constructed a detailed phylogenetic tree of the FH2 domains of nearly 300 formin sequences from our collection (Figure 1, Additional file 3).

We have successfully recovered all the major previously reported formin groups, namely the 7 metazoan classes (Fmn, FHOD, Frl, Daam, Delphilin, Dia, Inf), a compact group of yeast (i.e. ascomycete) formins [6], as well as plant Class I and Class II formins [7]. Noticeably, choanoflagellate (*Monosiga brevicollis*) FH2 domains tend to cluster together with animal ones in most cases. Three new clades containing FH2 domains of multiple organism have been recovered – namely two branches containing most (but not all) basidiomycete and zygomycete formins, a cluster containing the majority of ciliate formins, and a somewhat poorly supported cluster of adjacent branches containing exclusively stramenopile sequences (Figure 1). Nevertheless, many of protist formins could not be assigned to any of the previously defined classes; and also some algal formins fell into the unresolved deep branches together with the majority of protist sequences. This could perhaps be at least partially due to erosion of meaningful phylogenetic signal by a build-up of mutations, and possibly also to long branch-associated artifacts.

On the contrary, topologies of some of the well-defined branches, in particular the plant Class I and Class II formins where numerous sequences have been included in the analysis, as well as the FHOD cluster, indicate a relatively low degree of within-class divergence that suggests recent diversification (unless we assume major between-branch differences in selection pressure). It is thus likely that multiple duplications of formin-encoding genes took place independently in diverse lineages.

To our surprise, a third group of FH2 domains, possibly related to plant Class I but distinct from them, was found in some non-seed plants, namely in the lycophyte *Selaginella moelendorffii*, the moss *Physcomitrella *patens and in two prasinophyte algae (*Ostreococcus sp.*). Since this group exhibits also a specific domain organization (see below), we suggest recognizing these formins as a specific subgroup of plant FH2-containing proteins. We will further refer to these proteins as plant Class III formins.

### Diversity of formin domain architectures

While phylogenetic analysis based on FH2 domain sequences captures evolution driven by point mutations, it provides no information on domains outside FH2. We have therefore searched all complete formin sequences for a collection of known protein domains from the SMART and Pfam databases using the SMART search tool [28,29]. In addition, BLAST was used to search for homologues of the plant FH2-associated PTEN-like domain that is not included in the SMART/Pfam domain set [8]. Results are summarized in Table 1 (non-plant lineages), Table 2 (a more detailed view of plant formins) and in Additional files 4 and 5.

**Table 1:** Diversity of non-plant FH2 proteins

Kingdom	Phylum	FH1	GBD/FH3	DAD	PH	C2	PDZ	PTEN	tm	Other
**Animalia**	Chordata	+	+	+	-	-	+	-	-	-
	Nematoda	+	+	-	-	-	-	-	+	-
	Annelida	+	+	+	-	-	+	-	+	-
	Mollusca	+	+	+	-	-	+	-	-	-
	Insecta	+	+	+	-	-	-	-	-	-
	Crustacea	+	+	+	-	-	-	-	-	-
	Choanoflagellata	+	+	-	+	-	-	-	-	-
**Fungi**	Ascomycota	+	+	-	-	-	-	-	-	-
	Basidiomycota	+	+	-	-	-	-	-	-	-
	Zygomycota	+	+	-	-	-	-	-	-	-
**Amoebozoa**	Dictyostelida	+	+	-	-	+	-	-	-	-
	Entamoebidae	+	+	-	-	-	-	-	-	-
**Chromalveolata**	Apicomplexa	+	-	-	-	-	-	-	-	-
	Ciliophora	+	+	-	-	-	-	-	-	ARM
	Stramenopila	+	+	-	+	+	-	+	-	ANK, WW
**Excavata**	Kinetoplastida	+	-	-	-	-	-	-	-	-
	Parabasalia	+	+	-	-	-	-	-	-	-
	Heterolobosea	+	+	-	-	-	-	-	+	-
**Plantae**	See Table 2	+	-	-	-	-	-	+	+	RhoGAP

An overview of conserved domains and motifs in non-plant FH2 proteins. For each group of organisms, presence or absence of conserved domains or motifs found in formins of at least two species is indicated (if found in at least two proteins of only one lineage, they are listed as "other"); for more detailed table including single-instance domains, a full list of species and numbers of genes see Additional file 4. Domain abbreviations and accessions are: FH1 – formin homology 1 (a polyproline stretch detected visually), GBD/FH3 – GTPase binding domain of Diaphanous-related formins [Pfam: Drf_GBD] and/or FH3 domain of Diaphanous-related formins [Pfam: Drf_FH3], DAD – Diaphanous autoinhibitory domain [Pfam: Drf_DAD]; PH – pleckstrin homology domain [Smart: SM00233], ANK – ankyrin repeats [Smart: SM00248], C2 – protein kinase C conserved region 2 [Smart: SM00239], PDZ – domain present in PSD-95, Dlg, and ZO-1/2 [Smart: SM00228]; PTEN – phosphatase and tensin homology domain (identified by BLAST), tm – a secretion signal/transmembrane helix combination.

**Table 2:** Diversity and domain structure of plant FH2 proteins

Division	FH1	Class I	Class II	Class III	Other FH2	tm	PTEN	RhoGAP
Angiospermophyta	+	+	+	-	-	+	+	-
Lycophyta	+	+	+	+	-	+	+	+
Bryophyta	+	+	+	+	-	+	+	+
Chlorophyta	+	-	-	-	+	-	-	-
Prasinophyta	+	-	-	+	+	-	+	+
Rhodophyta	+	-	-	-	+	+	-	-

An overview of conserved domains and motifs in plant (archeplastid) FH2 proteins. Presence or absence of FH2 domains of the three plant-specific formin clades, as well as conserved domains or motifs found in formins of at least two species, are indicated. For a more detailed table including single-instance domains, a full list of species and numbers of genes see Additional file 5. RhoGAP – GTPase-activator protein for Rho-like GTPases [Smart: SM00324]; for remaining domain abbreviations see Table 1.

The majority of FH2 proteins analyzed appears to contain a rather limited selection of additional domains outside FH2, with the canonical GBD/FH3-FH1-FH2 set being the most frequent domain architecture. The C-terminal DAD motif usually found in GBD/FH3-containing formins is defined very stringently in the Pfam database, as it detects only a subset of proteins reported to contain it (namely formins of the Diaphanous subfamily) and misses, e.g., all yeast and *Dictyostelium *formins, as well as some mammalian ones. We will further refer to "GBD/FH3-FH1-FH2" architecture or "GBD/FH3-containing formins" in all cases where the presence of DAD could not be documented by statistically significant detection of the Pfam motif [Pfam: Drf_DAD], reserving the full "GBD/FH3-FH1-FH2-DAD" description to cases where this motif was found unambiguously. Nevertheless, we believe that presence of GBD/FH3 is likely to indicate regulation by means of GTPase-dependent release of intramolecular inhibition *via *a canonical or variant DAD that may be detected upon visual inspection [5] and data not shown). GBD/FH3-containing formins have been found in representatives of all kingdoms with the exception of plants, although only in animals and fungi they were present in all species; this is consistent with this domain architecture being ancestral.

Major eukaryotic lineages exhibit substantial differences in the degree of formin architecture diversity. Fungi represent one extreme; indeed, all fungal formins studied exhibited the standard GBD/FH3-FH1-FH2 combination, and no other conserved domains were identified. On the other end of the scale are some chromalveolate lineages, in particular the stramenopiles, and, to a lesser extent, also the metazoa, which have combined the FH2 domain with a diverse array of other conserved modules. Although such "exotic" formin structures have to be interpreted cautiously, since gene prediction artifacts cannot be excluded in the absence of experimentally determined cDNA sequences, at least in one case (the *Caenorhabditis elegans *FOZI-1 protein [30]), a protein containing FH2 in combination with a DNA binding (zinc finger) domain exhibited biological activity.

**Figure 2:**
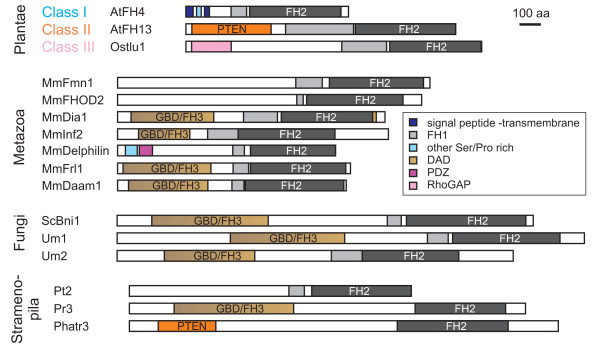
**Examples of formin domain organization**. Typical members of the three plant and seven metazoan formin classes are shown, compared to selected fungal and stramenopile formins (drawn to scale).

Some chromalveolate species, such as *Cryptosporidium parvum*, *Thalassiosira pseudonana *and *Phaeodactylum tricornutum*, as well as the excavates *Leishmania major *and *Trypanosoma sp*., lack GBD/FH3-containing formins altogether. However, plants represent the only kingdom that appears to have entirely disposed of the canonical structure. Instead, they possess up to three major groups of formins. Besides the previously described Class I (usually transmembrane) and Class II (PTEN-containing) formins characteristic for angiosperm plants [8], we found that the novel Class III formins of some non-seed plant species carry a N-terminal domain homologous to the Rho – associated GTPase activating protein (RhoGAP). We will discuss this domain, which appears to be specific to plant Class III formins, in more detail below. A schematic view of some of the observed formin domain organizations is presented in Figure 2.

### A novel RhoGAP-like domain in formins of non-seed plants

**Figure 3:**
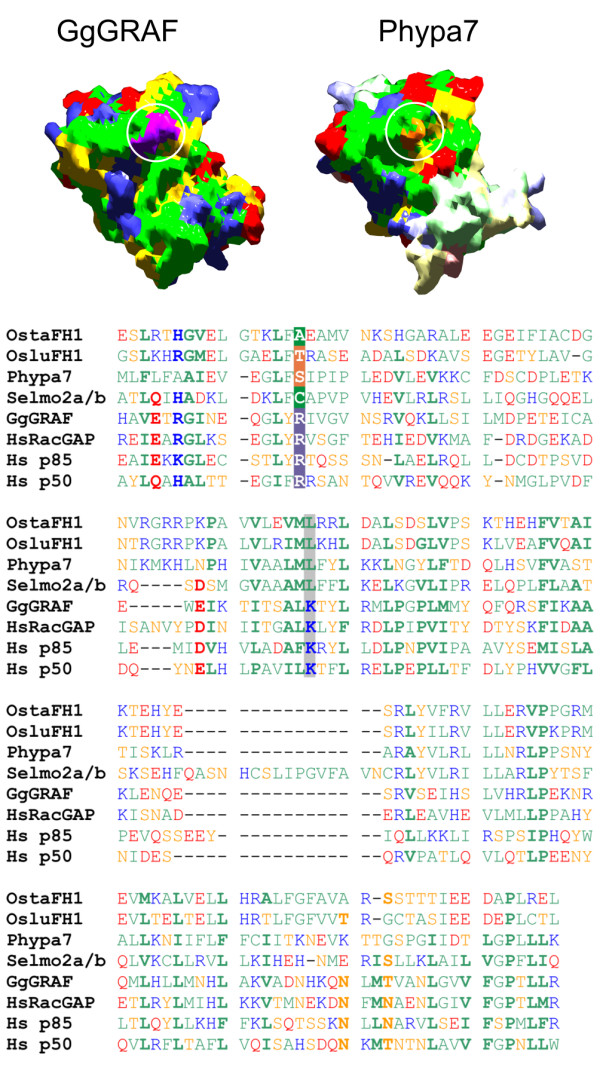
**The RhoGAP-related domain of Class III plant formins**. Top – comparison of the experimentally characterized structure of the mammalian BH (BCR homology) Rho GAP domain from the chicken protein GRAF (GgGRAF, [PDB: 1F7C]) and a three-dimensional model of the RhoGAP domain of the *Physcomitrella patens *Class III formin Phypa7, constructed as described in Materials and Methods. Surface residues are colored using the standard "color by type" scheme of SwissModel, except of the conserved arginine in the arginine finger of GgGRAF (purple) and the corresponding serine of Phypa7 (orange), also marked by a circle. Less confidently predicted parts of the model are shown in pale colors. Bottom – a structure-based alignment of the RhoGAP domains of four Class III formins with four structurally characterized mammalian Rho or Rac GAPs (GgGRAF – see above, HsRacGAP – human Rac-specific GAP/beta-chimaerin [PDB: 1XA6], Hs p85 – human RhoGAP-like BH-domain from phosphoinositide 3-kinase p85 [PDB: 1PBW], Hs p50 – the GTPase-activating domain from human p50rhoGAP [PDB: 1RGP]). Amino acids coloring as in the three-dimensional model, positions conserved across all mammalian sequences in bold, the arginine finger or corresponding diverged residues inverted (white on a coloured background), another similarly diverged site (conserved Lys to Leu mutation in formin-derived sequences) is shown on a gray background.

The novel, plant-specific combination of a Class III FH2 domain and a RhoGAP-related domain was found in four proteins: one from a lycophyte (Selmo2a/b), one from a moss (Phypa7) and one each from two prasinophytes (Ostta1 and Ostlu1). Another prasinophyte genome released after the submission of our manuscript (two clones of *Micromonas pusilla *sp.) also appears to encode at least one Class III formin upon brief inspection (see JGI Protein ID: 100127 and Protein ID: 47518). To our surprise, detailed examination of an alignment of the RhoGAP-related domain sequences revealed significant deviations from the common RhoGAP consensus, most notably replacement of the central arginine of the "arginine finger" [31,32] which appears to be involved in GTPase activation by Ras, Rho and Rab GAPs, by a small aliphatic or polar residue. Also a similarly conserved lysine residue downstream of the arginine finger appears to be replaced by leucine in all the plant sequences (Figure 3). A three-dimensional model of the RhoGAP-related domain constructed by threading of Phypa7 on known Rho or RacGAP domain structures (Additional file 6) indeed confirmed a local alteration in shape and charge of the conserved GTPase interaction interface, while the overall conformation of the molecule appears preserved. Thus, the RhoGAP-like domain of Class III thus might not function as a GTPase-activating protein, while its ability to bind a Rho-class GTPase may be retained. Nevertheless, since an alternative mechanism of GTPase activation involving an asparagine at a closely related position instead of the arginine finger has been described for Rap1GAP [33], we cannot exclude that the GAP domains of Class III formins also "invented" their own way of supporting GTPase activity.

Identification of RhoGAP-related domains in Class III formins of prasinophytes and two early land plants (the moss *Physcomitrella *and the lycophyte *Selaginella*) prompted us to search for closely related domains among the previously characterized members of the RhoGAP family. In phylogenetic trees based on a combination of Class III RhoGAP-domain sequences with a recently published set of human RhoGAPs [34], the RhoGAP-like domains from Class III formins cluster together, however their relation to any of the human RhoGAPs cannot be resolved (data not shown). A BLAST search of the non-redundant NCBI Entrez database with the Phypa7 RhoGAP domain as a query identifies predominantly metazoan (especially insect), fungal and *Dictyostelium *proteins with E-values in the range of 5.10^-9 ^to 10^-4^, the only exception being Ostta1. The remaining RhoGAP-like domains of Class III formins find no significant matches by BLAST; however, they produce results analogous to Phypa7 when the more sensitive PSI-BLAST algorithm [35] is used. We can thus conclude that the RhoGAP-like domains of Class III formins are more closely related to opisthokont RhoGAPs than to plant RopGAPs.

### PTEN-containing domains outside land plants

Several of the observed domain architectures were found in formins of multiple distantly related lineages. If multiple instances are found within one kingdom (e.g. in case of the PDZ domain in metazoans or the PH and ANK domains of stramenopiles), kingdom-specific acquisition followed by gene loss is the obvious explanation. However, some domains exhibit a discontinuous or punctate distribution across more than one kingdom. This is the case e.g. of the C2 domain (single instances in *Dictyostelium *and stramenopile formins), the PH domain (sole occurrences in two stramenopiles and the choanoflagellate *Monosiga brevicollis*), the PTEN domain (relatively common in stramenopiles and plants), and the secretion signal – transmembrane segment combination (common in plants, rare in invertebrates, two cases in the amplified formin family of *Naegleria*; outside plants, only the *Caenorhabditis *case is cDNA-supported). The first two domains occur in isolated instances, suggesting independent origins of the C2-FH2 and PH-FH2 combinations, while the small size and relatively loose sequence requirements for secretion and transmembrane signals points to possible convergent evolution. The case of PTEN is more complex, and deserves a detailed analysis.

The PTEN-related domain is characteristic for plant Class II formins, found in angiosperms, lycophytes and mosses. However, it was also identified in prasinophyte formins that cannot be reliably assigned to any of the three FH2 clades characteristic for land plants (see Figure 1, Table 2 and Additional file 5), as well as in formins of stramenopiles (namely diatoms and the plant parasite *Phytophtora sp.*). The relationships between stramenopiles and plants may be somewhat suggestive of horizontal gene transfer associated with either endosymbiosis or parasitism (in *Phytophtora*), although this can be neither confirmed nor rejected on the basis of the FH2 domain sequence since the relationship of stramenopile FH2 to the plant formins remains unresolved.

**Figure 4:**
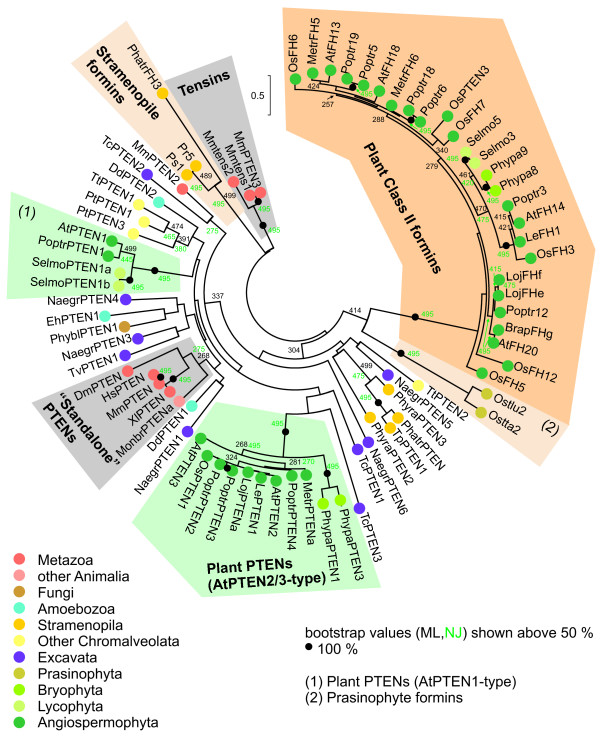
**Phylogeny of PTEN domains**. An unrooted maximum likelihood tree of 76 PTEN domains constructed as described in Materials and Methods. For a full list of genes, see Additional files 1 and 7. Only sequences that did not introduce large gaps into the alignment were chosen; for a tree from a larger selection of PTEN domains see Additional file 9. Numbers at nodes denote bootstrap values (out of 500 replicates; branches supported in all bootstraps marked by a dot). For comparison, bootstrap values from a NJ tree constructed on the basis of the same data are shown in green for major branches (from 500 bootstrap samples).

To obtain insight into possible origins of discontinuous phylogenetic distribution of the PTEN-FH2 architecture, we have assembled a collection of 63 "standalone" PTEN domain sequences (i.e. sequences of PTEN-containing proteins that do not carry the FH2 domain) from 30 species (see Additional files 7 and 8) and performed a phylogenetic analysis, including also 36 PTEN domains of the previously identified plant and stramenopile formins. After removing sequences whose inclusion would have introduced gaps that would lead to substantial shortening of the alignment, we obtained a tree (Figure 4), which exhibits a generally better statistic support than the FH2 domain tree and classifies the PTEN domains of prasinophyte formins as sister group of the PTEN domains of angiosperm Class II formins, while stramenopile FH2-associated PTEN domains form a separate clade. However, a tree based on a shorter alignment including more sequences (Additional file 9) swaps the position of the prasinophyte and stramenopile FH2-associated PTEN domains; thus we have to consider their relationship to angiosperm Class II formins still unresolved. Nevertheless, neither tree supports monophyletic ancestry of PTEN domains from prasinophyte and stramenopile formins, suggesting an independent origin of the PTEN-FH2 association rather than horizontal gene transfer.

**Figure 5:**
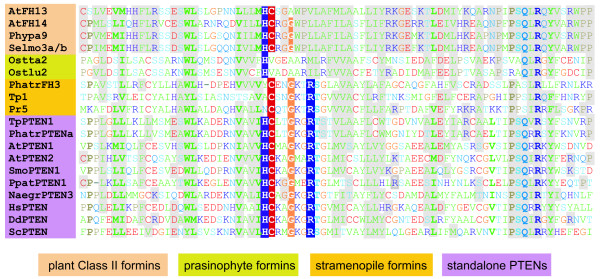
**The PTEN domain of plant class II formins, stramenopile formins and selected "standalone" PTENs**. Alignment of the central part of selected PTEN domains representing land plant Class II formins, PTEN-associated formins of prasinophytes and stramenopiles, and standalone PTENS from a range of eukaryotes (see Additional files 1 and 7 for sequence description and the color key in the figure for classification). Sequences are colored by residue type using the standard BioEdit coloring scheme. Positions conserved across all standalone PTENs are in bold, those shared by all Class II formins are on a gray background. The catalytic site consensus is inverted (white on coloured backgtround).

Independent acquisition of a PTEN-like domain in stramenopile formins is also supported by the finding that, similar to plant Class II formins [8], the prasinophyte, but not stramenopile, formins lack a crucial arginine residue in the conserved catalytic site of the PTEN protein/lipid phosphatase [36,37] and are thus unlikely to exhibit enzymatic activity. However, stramenopile formins have retained the canonical arginine residue (Figure 5). Nevertheless, they also probably lost the catalytic activity, since they are missing another conserved residue (the histidine within the conserved H-C-X-X-G-X-X-R signature motif), raising thus the possibility that stramenopiles not only independently invented the PTEN-FH2 domain architecture, but also found their own way to disable the enzyme activity of the PTEN domain.

## Discussion

Formins (FH2 domain-containing proteins) are an abundant family of actin-organizing proteins conserved across multiple major eukaryotic lineages. However, detailed phylogenetic analyses so far focused only on opisthokonts, *Dictyostelium *and angiosperm plants [2,5-8]. While the results provided a glimpse of the extent of formin domain architecture variability, little was known until now about FH2 protein diversity outside these lineages. An attempt to trace down the origins of the conserved parts of the actin-nucleating machinery, including formins, failed to identify significant relatives of the conserved FH2 domain among prokaryotes. Nevertheless, formins were found in all eukaryotes sampled so far (except *Giardia*), suggesting their presence already in the last common ancestor of eukaryotes [38]. In this study, we attempt to map the diversity of formins across most eukaryotic kingdoms, focusing not only on the FH2 domain itself but also on the overall domain architecture of the multi-domain FH2-containing proteins, and to reconstruct a possible evolutionary scenario that has produced the unique domain architectures found in formins of present-day plants.

The selection of species sampled was constrained mainly by public availability of searchable complete or draft genome sequences. We have included representatives of most major eukaryotic kingdoms [27,39]: animals (metazoans and choanoflagellates), several lineages of fungi, amoebozoa, chromalveolates, excavates and a diverse selection of plants. We could not include any representative of the Rhizaria because of lack of data. However, recent phylogenetic studies suggest that the rhizarians may, in fact, represent a branch of chromalveolates [40,41], which would mean that our collection covers all major eukaryotic lineages.

Since we focused on the origins of the domain architecture of plant formins, we sampled the plant lineage in more detail. Our analysis covers three complete genomes and several cDNA or EST collections from angiosperms, one moss genome, five algal genomes representing the chlorophytes, prasinophytes and rhodophytes, and a partial genome sequence from a lycophyte. However, data are still lacking from many lineages that may be crucial for filling the gaps in our evolutionary reconstruction. In particular, gymnosperm sequences might aid in pinpointing the disappearance of Class III formins that are present in mosses and lycophytes but absent in angiosperms, charophyte data would probably help to resolve the events associated with acquisition of the Class I (transmembrane) formin architecture characteristic for land plants, and glaucophyte data would provide insight into the early steps in the establishment of the plant lineage.

An initial database search revealed an astonishing diversity of formins in most eukaryotes. With the exception of some ascomycete fungi, all organisms studied have at least two formin-encoding genes, and the number exceeds a dozen in many lineages, including the unicellular flagellate amoeba *Naegleria*. Thus, the diversity of formins obviously does not reflect functional demands related to production of multiple cell types or complex cell-to-cell boundaries, as would be expected from observations of tissue-specific expression of some formins in metazoans [42-45] or plants [12] or from subcellular domain-specific localization of formin proteins in metazoan [46-48] and plant [13,14,49] cells. A recent large-scale analysis of 1219 protein superfamilies indicates that such a lack of correlation with the organisms' biological complexity (roughly quantifiable e.g. on the basis of estimates of the number of cell types) may represent a rather typical situation [50].

Nevertheless, highest numbers of formins among unicellular organisms were found in *Naegleria *and *Paramecium*, i.e. in organisms possessing rather sophisticated cell surface structures that might also bring specific requirements on (cortical) cytoskeletal organizers, including the FH2 proteins.

We have performed a detailed phylogenetic analysis of nearly 300 FH2 domain sequences (see Figure 1 and Additional file 3). Because of the relatively small size and high divergence of the FH2 domain, only limited resolution was achieved. Moreover, lack of a suitable outgroup prevented convincing characterization of mutual relationships between the well-defined branches. Nevertheless, we have confirmed the presence of the 7 previously proposed mammalian groups [6], and two classes in angiosperm plants [7]. However, the basal position and monophyly of fungal formins, originally suggested on the basis of a very limited set of data [6], was not confirmed, albeit FH2 domains of ascomycete yeasts did cluster together (but apart from basidiomycete and zygomycete ones, which together formed a separate branch). Even within the conserved clades, identification of orthologues is difficult or impossible. The topology of the well-resolved branches of the phylogenetic tree strongly suggests that they represent clusters of paralogues that have arisen by series of independent, species-specific gene duplication events. Besides of the previously known two plant formin classes, we have identified a novel formin clade present only in non-seed green plants (i.e. prasinophyte algae *Ostreococcus sp*., the moss *Physcomitrella patens*, and the lycophyte *Selaginella moelendorffii*), which we termed Class III.

Analysis of the domain architecture of our formin collection revealed several novel domain combinations besides the "canonical" opisthokont GBD/FH3-FH1-FH2 structure and the two architectures previously described for angiosperms (see Table 1 and 2, Figure 2 and Additional file 4 and 5). Some of these novel architectures have already been described either in the course of systematic surveys [2,5,8] or incidentally on the occasion of cDNA or gene cloning (e.g. the FH1-less *Dictyostelium *formin ForC [51], or the *Caenorhabditis *FOZI-1, which carries a zinc finger motif [30]).

In particular, the association between the PTEN domain (occurring in plant Class II formins and some stramenopile ones) exhibited a discontinuous (or punctate *sensu *[52]) distribution across the eukaryotic evolutionary tree, indicating either two independent gene fusion events, repeated losses or horizontal gene transfer. Independent origin of identical domain combinations is considered extremely rare and thus unlikely [53]. However, this conclusion was based on an analysis of a sample of 57 prokaryotic genomes and only 5 eukaryotic ones, and its validity for eukaryotes may be therefore questionable. Since the PTEN domains of stramenopile formins do not form a sister group to those of plant Class II formins (Figure 4), we indeed suspect that the association between the PTEN and FH2 domains was established twice. Moreover, independent association with the FH2 domain was in both cases accompanied by an obvious loss of a conserved catalytic site, which was accomplished by different means in each lineage, i.e. again in a convergent fashion (Figure 5).

Mutational inactivation of conserved, originally biochemically active domains might indeed present a common theme in formin evolution. We found that all formins with Class III FH2 domains posses a N-terminal domain exhibiting considerable similarity to the Rho GTPase-activating protein (RhoGAP) family proteins. However, the conserved arginine finger, essential for stimulation of the Rho GTPase activity by RhoGAPs [32,54], appears to be eliminated by mutations in all cases. Thus, the RhoGAP-like domain of plant Class III formins is unlikely to act as a GTPase activating protein, unless it acquired the ability to activate GTPases through a different mechanism, as described in the case of the mammalian Rap1GAP [33]. "Inactive" members of the RhoGAP family have been identified previously, such as mammalian p85, which lost the GAP activity while retaining its arginine finger, ARAP2 that lacks the arginine finger [55], or mammalian and fungal IQGAPs [56,57]. We believe that although proteins with GAP domains are generally viewed predominantly as functionally well-defined components of the GTPase functional cycle, at least some of them obviously have acquired additional functions besides facilitating GTP hydrolysis and thus "re-setting" or "erasing" a signal carried by a GTP-loaded or "active" GTPase. Obviously, to facilitate GTP hydrolysis, GAPs have to interact with GTP-loaded GTPases, becoming thus also prime candidates for their effectors. In case of plant Class III formins, ARAP2, IQGAP or p85, this presumed effector function apparently became selectively advantageous enough to allow surviving subsequent loss of the GAP activity. Indeed, IQGAPs have been originally discovered as effectors of Rho family GTPases, especially Cdc42 [56,57].

The Rho GTPases are peripheral membrane proteins well-known for their participation in the control of cell surface-associated actin cytoskeleton, contributing to the formation of structures as diverse as lamellipodia and filopodia of animal cells, yeast buds, and plant root hairs and/or pollen tubes (for a review see [38,58-60]. IQGAPs mediate Rho GTPase-based control of actin organization at the leading edge and cell-to-cell junctions of mammalian cells [56,57,61]. The C-terminal inactive GAP domain of IQGAP binds to activated Rho and functions as a GAP inhibitor [56], while the N-terminal calponin domain interacts with the F-actin, effectively stimulating F-actin accumulation at the activated Rho cell-cell contact junctions [62,63].

**Figure 6:**
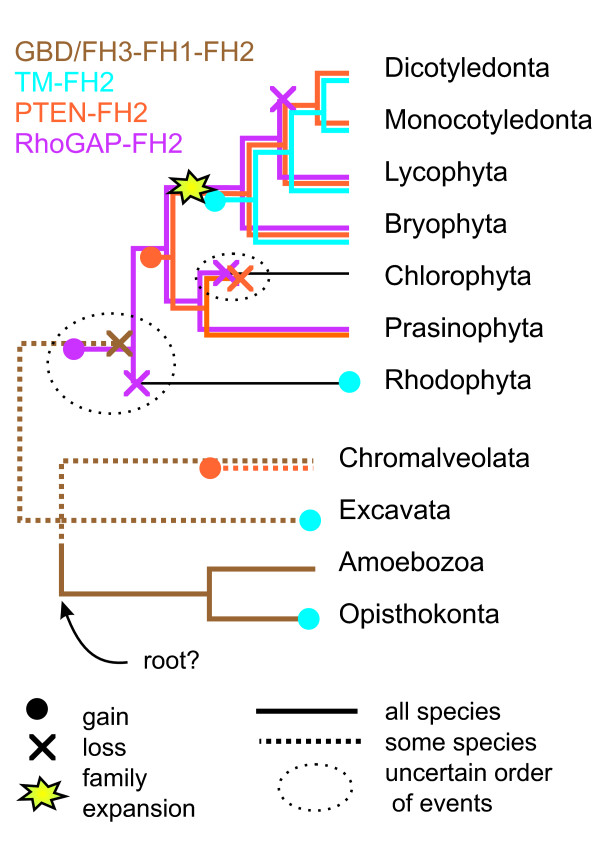
**A possible scenario of formin evolution**. Distribution of formin domain architectures mapped on a phylogenetic tree of selected eukaryotic lineages, with focus on plants. Typical domain architectures are color-coded: GBD/FH3-FH1-FH2 in brown, transmembrane (plant Class I-like) in blue, PTEN-associated (plant Class II-like) in orange, plant Class III-like in purple. Phylogeny based on [27,114,115].

The conserved domain architecture of formins carrying a GTPase-binding domain (GBD/FH3), believed to mediate Rho-dependent activation of the formin through releasing intramolecular inhibition, stresses out the ancestral nature of another connection between Rho and actin organization – namely formin-mediated actin nucleation [5]. However, plants have apparently lost the canonical GBD/FH3-FH1-FH2 architecture. Nevertheless, our present analysis of the phylogenetic distribution of formin domain architectures in the plant lineage suggests that the ability to interact with a Rho type GTPase presents a crucial conserved feature of many (though not all) formins even in plants. In particular, the combination of a presumably inactive RhoGAP-like domain with an F-actin interacting domain in early land plant formins is reminiscent of IQGAPs, despite opposite domain order (the GAP-related domain is N-terminal in formins and C-terminal in IQGAPs), suggesting a remarkable convergence also on the functional level. We suggest that the extant common architectures of plant Class I, Class II and Class III formins may have been produced by a relatively simple sequence of evolutionary events that has maintained a continuity of the Rho-FH2 and/or membrane-FH2 association, while the molecular mechanisms of these connections have changed (Figure 6).

First, the ancestral GBD/FH3 domain was replaced by a probably catalytically inactive RhoGAP-like domain at some point prior to the divergence of algae (prasinophytes and chlorophytes) and the lineage leading to extant vascular plants. This event was concomitant with the emergence of Class III formins. We can only speculate about the situation in Rhodophyta since the only genome analyzed, that of *Cyanidioschyzon merolae*, represents an extremely derived, fast-evolving group [64] that either lost Class III formins or never possessed them (and disposed of the GBD/FH3 architecture independently). With respect to rhodophytes, the scenario shown in Figure 6 thus presents one of two equally parsimonious possibilities that maintain the Rho-FH2 connection; an alternative would involve replacement of GBD/FH3 by the RhoGAP-like domain only after the divergence of red algae, and loss of GBD/FH3 in the rhodophyte lineage.

In the "green" lineage leading towards green algae and vascular plants, a domain fusion produced the combination of FH2 with a catalytically inactive PTEN-related domain, generating thus the typical Class II architecture and providing a possible alternative mechanism for attachment of FH2 to a membrane. It is worth noting that the only chlorophytes lacking both Class II and Class III type architectures are the volvocal algae. We believe that this may again be a derived feature, since the recently published *Chlamydomonas *genome sequence [65] reveals that this organism has apparently lost Rho-type GTPases altogether, possibly together with the whole associated molecular apparatus.

Finally, in the vascular plant lineage a series of gene duplications took place in the formin gene family, starting prior to the divergence of Bryophyta but continuing later on. Amplified formin genes provided material for subsequent domain shuffling that produced the rich diversity of formins in extant angiosperms. It is tempting to speculate that this diversification may have been coincident with the ascent of plants on dry land. One of the innovations that appeared at the same time was the emergence of membrane-anchored Class I formins. Once the Class I and Class II formins became established, plants acquired Rho-independent means for associating the FH2 domains with membranes, which enabled subsequent loss of Class III formins in the angiosperm lineage.

In the course of our study, we have noticed that the diversity of formin domain architectures varies substantially among major eukaryotic lineages (Table 1 and 2, Additional data file 4 and 5), suggesting a variety of possible regulatory inputs controlling the activity of the FH2 domain, its localization and interactions with diverse intracellular structures. Despite of their rich formin gene families, neither plants nor metazoans present an extreme of FH2 domain mobility – the most diverse and variable formin architectures in our collection were apparently those of chromalveolates, mainly stramenopiles (while the other extreme is provided by ascomycete fungi). However, major differences in formin diversity are not restricted to the level of kingdoms (even if we take into account possible gene structure mispredictions). In particular, invertebrate metazoans, such as molluscs or *Caenorhabditis*, possess unusual domain combinations, such as formins carrying a membrane insertion signal or a zinc finger [30].

This leads to the open question what determines the varying degree of FH2 domain mobility, or promiscuity, in diverse lineages. Apparently this is not a feature of the FH2 domain per se, since all fungal, insect, crustacean and kinetoplastide formins studied so far exhibit only the canonical GBD/FH3-FH1-FH2 architecture or its even simpler FH1-FH2 variant. Alternatively, we could imagine that some lineages have a general tendency to amplify genes and generate novel domain combinations; thus, organisms with unusual formin architectures should also exhibit greater diversity in other modular, multidomain proteins. However, this is not the case – *Caenorhabditis elegans *has one of the richest families of formins among animals, but at least one other modular domain, namely SH2, exhibits rather modest diversity compared to other metazoans (Tony Pawson, personal communication). Indeed, results of a large-scale analysis of eukaryotic proteins containing domains from the Pfam database suggested that protein domains form lineage-rather than domain-specific combinations [66]. Although the authors of the cited study consider this an animal-specific feature, their conclusion is based on a data set containing mostly animal and fungal genomes, and only very few species from other lineages. In particular, no stramenopiles have been included, and plants were represented only by *Arabidopsis*, rice and the highly derived rhodophyte *Cyanidioschyzon merolae*. Upon closer inspection of the data from [66], mere omission of *C. merolae *would lead to the result that plants (i.e. *Arabidopsis *and rice) have a number of lineage-specific domain combinations comparable to that of mammals and birds.

Results of our analysis of the formin structure in plants and other eukaryotes thus support the conclusion that domain architecture variability is not only a lineage-specific characteristic, but also a feature of the protein family involved. On a more general level, they also point out the limits for extrapolation from lineage-biased data, which, after all, only provide information about the species analyzed.

## Conclusion

Our analysis of a set of nearly 300 formin sequences from a variety of species representing most of the eukaryotic diversity revealed a surprising variability in terms of gene number and domain composition both among and within individual lineages. Apart of ancestral domain combinations, such as the ubiquitous FH1-FH2 or the nearly ubiquitous GBD/FH3-FH1-FH2 architecture, lineage-specific domain architectures were found in particular in the plant lineage, where we identified a novel class of formins carrying a presumably catalytically inactive variant of the conserved RhoGAP domain. At least in case of the FH2 domain-containing proteins, we can conclude that the selection or "space" of available domain combinations is determined not only by the participating domains themselves, but also (or even predominantly) by the organismal lineage. Nevertheless, we were able to detect cases of repeated independent emergence of domain combinations, such as PTEN-FH1-FH2 in plants and stramenopiles, suggesting that there may be a certain preference of domain partnerships. In any case, we can conclude that the frequency of convergent domain combinations may be higher than expected before on the basis of predominantly prokaryotic data.

## Methods

### Sequence data sources

Searches for formin and PTEN gene or protein sequences were conducted in the following publicly available databases: the National Center for Biotechnology Information Entrez main database and HTGS sections (Entrez, [67]) for *Brassica rapa*, *Medicago truncatula*, *Lotus japonicus*, *Vitis vinifera*, *Nicotiana tabacum*, *Lycopersicon esculentum*, *Ostreococcus tauri*, *Mus musculus*, *Caenorhabditis elegans*, *Drosophila melanogaster*, *Saccharomyces cerevisiae*, *Schizosaccharomyces pombe*, *Ustilago maydis*, *Dictyostelium discoideum*, *Entamoeba histolytica*, *Cryptosporidium parvum*, *Plasmodium falciparum*, *Trypanosoma brucei*, *Trypanosoma cruzi*; the US Department of Energy Joint Genome Institute (JGI, [68]) for *Chlamydomonas reinhardtii*, *Ciona intestinalis*, *Daphnia pulex*, *Helobdella robusta*, *Laccaria bicolor*, *Lottia gigantea*, *Monosiga brevicollis*, *Mycosphaerella fijiensis*, *Naegleria gruberi*, *Ostreococcus lucimarinus*, *Phaeodactylum tricornutum*, *Phycomyces blakesleeanus*, *Physcomitrella patens ssp patens*, *Phytophthora ramorum*, *Phytophthora sojae*, *Populus trichocarpa*, *Postia placenta*, *Thalassiosira pseudonana *and *Volvox carteri*; The Arabidopsis Information Resource (TAIR, [69,70]) for *Arabidopsis thaliana*; the Genoscope French National Sequencing Center database (Genoscope, [71]) for *Vitis vinifera*; the Selaginella Genomics database (SG, [72]) for *Selaginella moellendorffii*; the Rice Annotation Project Database (RAP-DB, [73,74]) for *Oryza sativa*; the Cyanidioschyzon merolae Genome Project (CmGP, [75,76]) for *Cyanidioschyzon merolae*; The Sanger Institute GeneDB database (GeneDB, [77]) for *Leishmania major *(data kindly provided by the Sanger Institute/EULEISH, Seattle Biomedical Research Institute and FMRP sequencing centres); the ParameciumDB database (ParameciumDB, [78]) for *Paramecium tetraurelia*; the Tetrahymena genome database (TGD, [79,80]) for *Tetrahymena thermophila*; the J. Craig Venter Institute, formerly The Institute for Genomic Research (TIGR,[81]) for *Trichomonas vaginalis *and a some plant sequences (see Additional file 1).

### Identification of FH2 and PTEN-containing proteins

Genes encoding putative formin or PTEN homologues have been identified by BLASTP and TBLASTN searches [35,82] of the above databases, first using previously characterized members of the family [8,26] as the query, then with the most diverged sequences from the first round as the query, until no new significant matches appeared. In addition, annotation searches using the keywords "actin-binding FH2" have been used, in particular in the case of the JGI protein predictions. Presence of FH2 domains in predicted candidate open reading frames has been confirmed by a SMART search [28,83] for all genes. Final database checks have been done between September and November 2007.

### Revision of protein sequence predictions

Gene predictions have been checked and, if necessary, corrected essentially as described previously [8], based on information from algorithmic splice site predictions, EST or cDNA sequences, as well as comparison with closest homologous proteins. A cDNA prediction was accepted if it was supported by experimental data and/or multiple prediction methods; if possible, a variant closest to the consensus of the conserved domain(s) has been used. In several cases we modified the proposed protein sequence or even joined two neighboring ORFs previously predicted as separate genes. All the newly predicted or modified protein sequences are listed in Additional file 2.

For each new gene, splice sites were predicted using at least three of the following eukaryotic gene-prediction programs: GeneMark.hmm [84], FGENESH [85] at the Softberry, Inc. website [86], TWINSCAN [87], GeneScan [88] and GENEWISE [89]. FGENESH with similarities (FGENESH +) at [86] was used if the genomic sequence quality was poor or if other programs failed to predict a protein containing the FH2 domain, although a BLAST search revealed a significant match. MACAW [90] was used to map the resulting predictions, as well as EST or cDNA sequences found by BLAST (if available), to the genomic sequence. Utilities of the Sequence manipulation suite [91] have been employed for routine sequence manipulations, such as assembly of predicted ORF sequences or translation.

### Domain architecture analysis

SMART version 5.1 [28,29] has been used to search for conserved domains as described previously [8]. These searches were complemented by direct Pfam 22.0 database searches [92,93] that revealed additional matches, in particular for the GBD/FH3 and RhoGAP domains. Secretory signals and transmembrane segments have been verified using SignalP and TMHMM [94-96] on the CBS Prediction server [97].

### Sequence alignments

Preliminary alignments used e.g, for identification of possible missing exons (see above) have been constructed using the very fast and reliable Kalign program [98,99]. Final multiple sequence alignments for phylogenetic purposes were constructed using PROBCONS [100] with default parameters and manually edited in BioEdit [101] in order to remove short blocks and gap, as well as increase aligned amino acid similarity, as visually judged with the aid of a BLOSUM62-derived color code and consensus shading. Prior to phylogenetic tree calculation, non-aligned ends have been trimmed and all portions of the alignment where a substantial number of sequences contained gaps have been removed.

For the construction of Figure 3, DeepView [102] was used to align the four known RhoGAP structures together with the computed three-dimensional model of the Phypa7 RhoGAP domain. Corresponding structure-based sequence alignment was exported into a text file, manually converted to the FASTA format and imported into BioEdit [101]. The remaining three Class III formin RhoGAP sequences were then added and aligned manually, using a Kalign-generated alignment for reference.

### Phylogenetic analyses

Phylogenetic trees were build on the basis of multiple alignments constructed as described above using two different algorithms: a heuristic approximation of the maximum likelihood (ML) method as implemented in the PHYML program[103] and the neighbor-joining (NJ) method using the software package MEGA [104], which was also employed for bootstrap sample derivation and graphical representation of the resulting trees. In both cases, the JTT amino acid substitution matrix was used, and statistical significance of the result was estimated using bootstrap analysis with 100 to 500 samples.

### 3D model construction

An initial search for suitable threading templates for three plant RhoGAP sequences (Selmo2a/b, Phypa7 and Ostta1) performed using the ExPDB function of SwissModel [105,106] yielded no reliable templates. Two additional methods were thus used: CPH Models [107] and QuickPhyre, a web implementation of Phyre [108,109], which also computes a 3D model for the best templates found. In case of Phypa7, both approaches yielded significant hits corresponding to known Rho- or RacGAP domains (PDB:1XA6 by CPH; PDB:1F7C, 1PBW, 1RGP, 1TX4 and 1XA6 with 100% confidence and E-values of 10^-7 ^or better). Since the results were substantially worse for the remaining plant sequences, we chose Phypa7 for model construction using SwissModel, based on an alignment of templates found by the Phyre algorithm. Although the WhatCheck output provided by SwissModel reported some protein backbone problems, the resulting model appeared to be very similar to models generated by Phyre on all the highly probable templates. In particular, 3D alignment in the DeepView environment [110] revealed that the area of the arginine finger appears to be nearly identical in all models, while peripheral parts of the molecule somewhat diverged (shown in pale colour in Figure 3). Moreover, WhatCheck control [111-113], performed separately for those parts of the molecule where the models agreed, revealed similar problems in the templates themselves. We thus believe that our model reflects reasonably well the conformation around the conserved GTPase interaction interface.

## List of abbreviations

ANK: ankyrin repeats; BLAST: Basic local alignment search tool; C2: protein kinase C conserved region 2; DAD: Diaphanous autoregulatory domain; FH1: formin homology domain 1; FH2: formin homology domain 2; FH3: formin homology domain 3; FOZI-1: formin homology and zinc finger protein 1; GBD: GTPase binding domain; PDZ: domain present in PSD-95, Dlg, and ZO-1/2; PH: pleckstrin homology domain; PTEN: phosphatase and tensin homolog; RhoGAP: Rho GTPase activating protein. Abbreviations of protein domains mentioned only once are explained in the text or figure/table legends; for abbreviations of species see Additional file 1.

## Authors' contributions

MG performed the majority of database searches and all phylogenetic analyses and wrote parts of the manuscript. VŽ contributed to data interpretation and to the analysis of the RhoGAP-related domain and participated in writing the manuscript. FC participated in searching the databases, constructed the 3D model and drafted the manuscript.

## Supplementary Material

Additional file 1 **Summary of FH2 proteins analyzed in this study (MG_A1.xls)**. Full list of taxa and FH2 protein sequences included in this study, including an overview of protein domain structure. Database source and accession code is included for every sequence (for database abbreviations see Materials and Methods). For cases where gene structure prediction has been modified, newly predicted protein sequences are provided in Additional file 2. For abbreviations and SMART or Pfam identifiers of additional conserved domains found in the predicted proteins see legends to Tables 1 and 2 and Additional files 4 and 5; sp – signal peptide, tm – transmembrane domain; NA – not available (usually due to incomplete sequence). Format: MS Excel (*.xls). Click here for file

Additional file 2 **Predicted protein sequences of selected formins (MG_A2.txt)**. Protein sequences corresponding to genes listed in Additional file 1 whose prediction has been modified compared to the original database versions. Format: FASTA, raw text (*.txt). Click here for file

Additional file 3 **Phylogenetic tree of the FH2 domains (MG_A3.pdf)**. An unrooted maximum likelihood tree constructed as described in Materials and Methods. For a full list of list of genes, see Additional file 1. Numbers at nodes denote bootstrap values (out of 500 replicates; branches supported in all bootstraps marked by a dot). For comparison, bootstrap values from a NJ tree constructed on the basis of the same data are shown in green for major branches (from 100 bootstrap samples). Format: Adobe portable document (*.pdf). Click here for file

Additional file 4 **Diversity and domain structure of non-plant FH2 proteins (MG_A4.pdf)**. For each species, total number of FH2 domain-containing proteins (formins) is given, together with numbers of formins carrying any of the additional listed domains or motifs. Complete and draft genomes are shown in bold. For abbreviations and database accessions of domains found in more than one species see legend to Tab.1; domains found only in a single species (denoted as "other") are: DEP – domain found in Dishevelled, Egl-10, and Pleckstrin [Smart: SM00049], ZnF – zinc finger [Smart: SM00355], PHD – PHD Zinc Finger [Smart: SM00249], HDAC_int – histone deacetylase interacting domain [Smart: SM00761], BROMO [Smart: SM00297], C1 [Smart: SM00109], FHA – Forkhead-associated [Smart: SM00240], TPR – tetratricopeptide repeats [Smart: SM00028], ARM – Armadillo/beta-catenin repeats [Smart: SM00185], 1i84 – contractile protein [PDB: 1i84], KISc – kinesin motor [Smart: SM00129], SAP [Smart: SM00513], FYVE [Smart: SM00064], PAN_1 [Pfam: PAN_1], WW – a conserved domain containing two tryptophan residues [Smart: SM00456]. For commonly appearing domains, the table is color-coded as follows: green – present in all formins of the species, yellow – present in some formins, orange – absent, grey – incomplete data. Format: Adobe portable document (*.pdf). Click here for file

Additional file 5 **Diversity and domain structure of plant FH2 proteins (MG_A5.pdf)**. For each species, the total number of complete formin sequences is given as FH2, and the number of incomplete sequences that did not allow conclusive analysis of domain architecture is as FH2 (partial). Class I, Class II, Class III and Other FH2 denote numbers of formins carrying FH2 domains from the respective clades (or unassigned ones); the remaining abbreviations denote additional domains and motifs found in plant formins: Sec10 – Exocyst complex component Sec10 [Pfam: Sec10]; for remaining domain abbreviations and database accessions see legend to Table 1 and Table 2. Complete and draft genomes are shown in bold. For commonly appearing domains, the table is color-coded as follows: green – present in all formins of the species (for FH1) or of all characterized formins of the corresponding class (for remaining domains in color), yellow – present in some formins, blue – present in a formin that cannot be assigned to the corresponding class, orange – absent, grey – incomplete data. Format: Adobe portable document (*.pdf). Click here for file

Additional file 6 **Model of the RhoGAP-related domain from Phypa7 (MG_Phypa7.pdb)**. Coordinates of the model in the PDB format (*. pdb) which can be viewed using e.g. the free Deep View Viewer [110]. Click here for file

Additional file 7 **Summary of PTEN domains analyzed in this study (MG_A7.xls)**. List of taxa and PTEN protein sequences included in this study, in addition to FH2 – containing sequences that are listed in Additional file 1. Database source and accession code is included for every sequence; for database source abbreviations see legend to Additional file 1. For abbreviations and SMART or Pfam identifiers of additional conserved domains found in the predicted proteins see legends to Tables 1 and 2 and Additional files 1, 4 and 5; additional domains are: ABC-tran – ATP-binding casette transporter [Pfam:PF00005], MYSc – myosin/large ATPase [Smart: SM00242], SH2 – Src homology 2 domain [Smart: SM00252], PTB – phosphotyrosine-binding domain [Smart: SM00462], LIM – Zinc-binding domain present in Lin-11, Isl-1 and Mec-3 [Smart: SM00132]. For cases where gene structure prediction has been modified, new predicted protein sequences are provided in Additional file 8. Format: MS Excel (*.xls). Click here for file

Additional file 8 **Predicted protein sequences of selected PTEN-containing proteins (MG_A8.txt)**. Modified protein sequence predictions corresponding to some of the genes listed in Additional file 7. Format: FASTA, raw text (*.txt). Click here for file

Additional file 9 **Phylogenetic tree of 90 PTEN domains (MG_A9.pdf)**. An unrooted maximum likelihood tree of 90 PTEN domains constructed as described in Materials and Methods. For a full list of list of genes, see Additional file 7. Note that this tree is based on more sequences but a shorter alignment than that from Figure 3. For the 5 PTEN sequences not represented in any of the trees (Figure 3 or Additional file 9) due to close relationship to another PTEN or identification only during the final database checks, closest relatives are shown in Additional file 7. Numbers at nodes denote bootstrap values (out of 500 replicates; branches supported in all bootstraps marked by a dot). For comparison, bootstrap values from a NJ tree constructed on the basis of the same data are shown in green for major branches (from 500 bootstrap samples). Format: Adobe portable document (*.pdf). Click here for file
