# Supplementary figures and images for: Roots of angiosperm formins: The evolutionary history of plant FH2 domain-containing proteins
Source: BMC Evol Biol. 2008 Apr 22;8:115. doi: 10.1186/1471-2148-8-115 (PMC2386819; doi:10.1186/1471-2148-8-115)

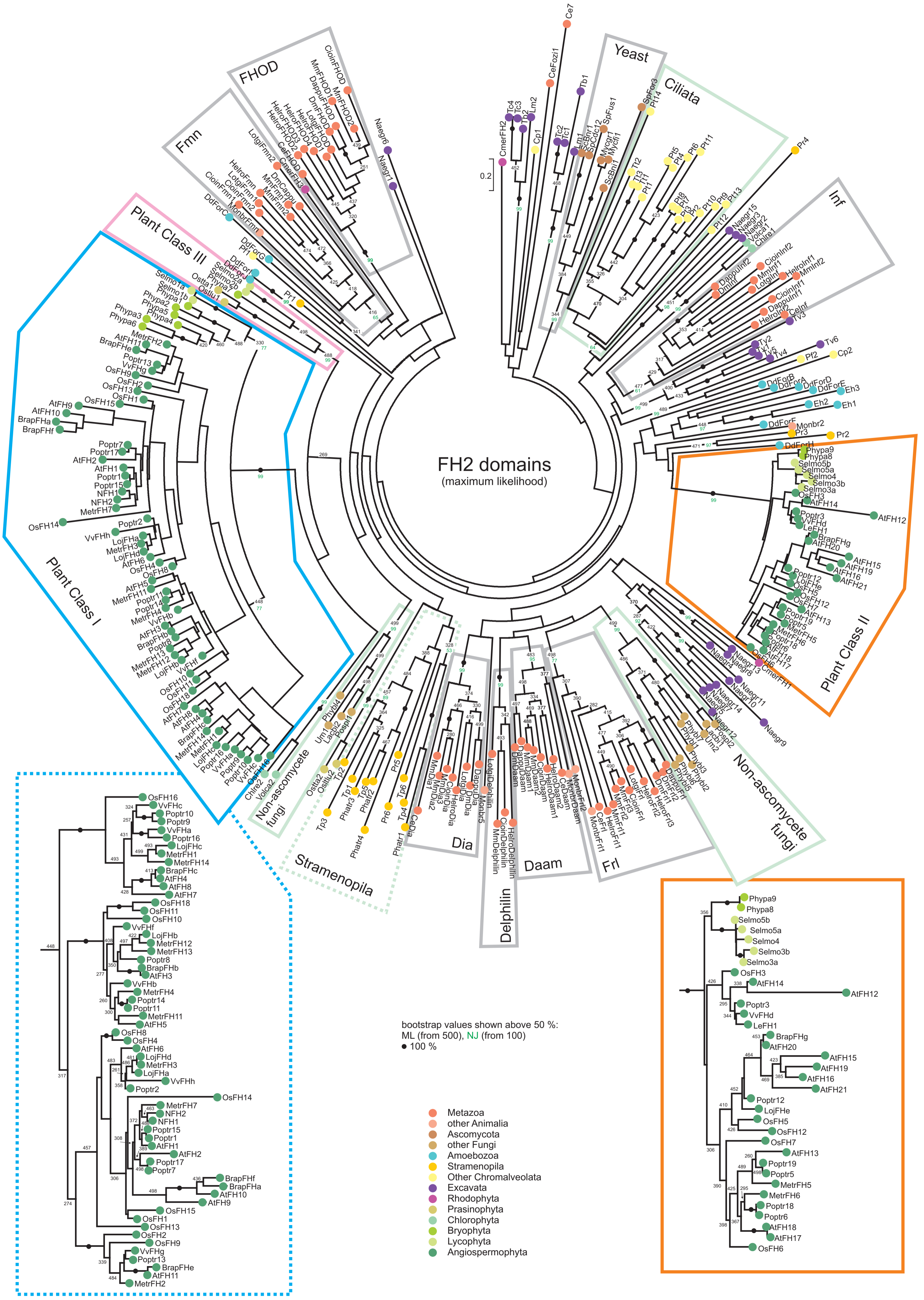

Supplement: Additional file 3 — Phylogenetic tree of the FH2 domains (MG_A3.pdf). An unrooted maximum likelihood tree constructed as described in Materials and Methods. For a full list of list of genes, see Additional file 1. Numbers at nodes denote bootstrap values (out of 500 replicates; branches supported in all bootstraps marked by a dot). For comparison, bootstrap values from a NJ tree constructed on the basis of the same data are shown in green for major branches (from 100 bootstrap samples). Format: Adobe portable document (*.pdf). [file 1471-2148-8-115-S3.pdf]

# PTEN domains

(maximum likelihood)

bootstrap values shown above 50 %:  
ML, NJ (from 500)  
● 100 %

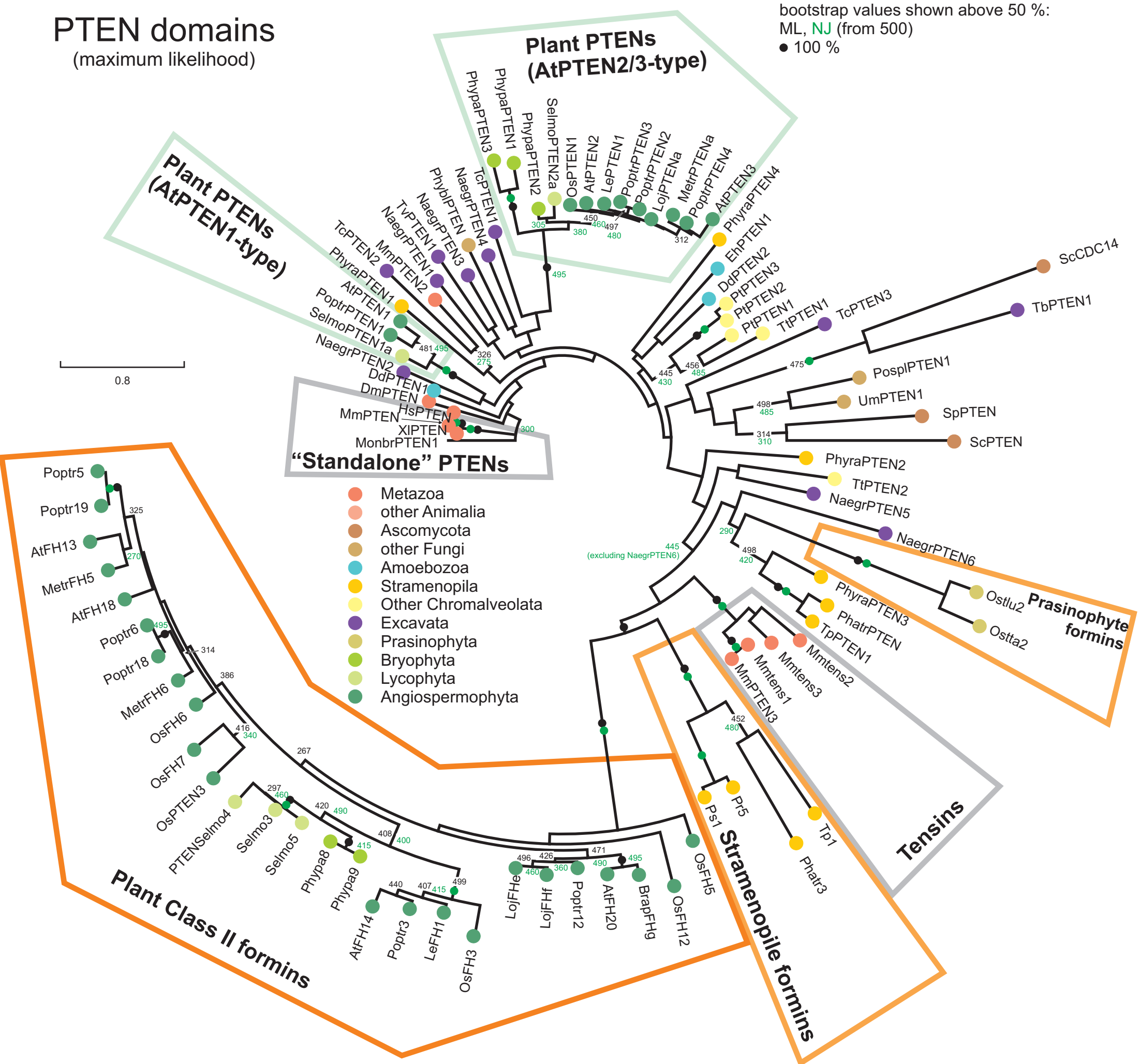

Supplement: Additional file 9 — Phylogenetic tree of 90 PTEN domains (MG_A9.pdf). An unrooted maximum likelihood tree of 90 PTEN domains constructed as described in Materials and Methods. For a full list of list of genes, see Additional file 7. Note that this tree is based on more sequences but a shorter alignment than that from Figure 3. For the 5 PTEN sequences not represented in any of the trees (Figure 3 or Additional file 9) due to close relationship to another PTEN or identification only during the final database checks, closest relatives are shown in Additional file 7. Numbers at nodes denote bootstrap values (out of 500 replicates; branches supported in all bootstraps marked by a dot). For comparison, bootstrap values from a NJ tree constructed on the basis of the same data are shown in green for major branches (from 500 bootstrap samples). Format: Adobe portable document (*.pdf). [file 1471-2148-8-115-S9.pdf]
