# Supplementary material for: Roots of angiosperm formins: The evolutionary history of plant FH2 domain-containing proteins
Source: BMC Evol Biol. 2008 Apr 22;8:115. doi: 10.1186/1471-2148-8-115 (PMC2386819; doi:10.1186/1471-2148-8-115)
Supplement: Additional file 5 — Diversity and domain structure of plant FH2 proteins (MG_A5.pdf). For each species, the total number of complete formin sequences is given as FH2, and the number of incomplete sequences that did not allow conclusive analysis of domain architecture is as FH2 (partial). Class I, Class II, Class III and Other FH2 denote numbers of formins carrying FH2 domains from the respective clades (or unassigned ones); the remaining abbreviations denote additional domains and motifs found in plant formins: Sec10 – Exocyst complex component Sec10 [Pfam: Sec10]; for remaining domain abbreviations and database accessions see legend to Table 1 and Table 2. Complete and draft genomes are shown in bold. For commonly appearing domains, the table is color-coded as follows: green – present in all formins of the species (for FH1) or of all characterized formins of the corresponding class (for remaining domains in color), yellow – present in some formins, blue – present in a formin that cannot be assigned to the corresponding class, orange – absent, grey – incomplete data. Format: Adobe portable document (*.pdf). [file 1471-2148-8-115-S5.pdf]

Division	Species	FH2	FH2 (partial)	FH1	Class I FH2	Class II FH2	Class III FH2	Other FH2	TM	PTEN	RhoGAP	Other
Angiospermophyta	**Arabidopsis thaliana**	21	-	20	11	10	-	-	10	4	-	-
	**Populus trichocarpa**	18	1	18	12	6	-	-	12	6	-	-
	Brassica rapa	6	-	6	5	1	-	-	5	1	-	-
	Medicago truncatula	10	1	8	8	2	-	-	8	2	-	-
	Lotus japonicus	6	1	6	4	2	-	-	4	2	-	-
	Vitis vinifera	7	1	6	6	1	-	-	6	1	-	-
	Nicotiana tabacum	2	-	2	2	-	-	-	2	-	-	-
	Lycopersicon esculentum	1	-	1	-	1	-	-	-	1	-	-
	**Oryza sativa**	17	-	17	12	5	-	-	12	5	-	-
Lycophyta	Selaginella moelendorffii	10	-	9	2	6	2	-	2	6	2	-
Bryophyta	**Physcomitrella patens**	9	-	7	6	2	1	-	5	2	1	Sec10
Chlorophyta	**Chlamydomonas reinhardtii**	2	-	1	-	-	-	2	-	-	-	-
	**Volvox carteri**	2	-	2	-	-	-	2	-	-	-	-
Prasinophyta	**Ostreococcus tauri**	2	-	2	-	-	1	1	-	1	1	-
	**Ostreococcus lucimarinus**	2	-	2	-	-	1	1	-	1	1	-
Rhodophyta	**Cyanidioschyzon merolae**	3	-	2	-	-	-	3	1	-	-	-
